# Does Pelvic Tilt Angle Influence the Isokinetic Strength of the Hip and Knee Flexors and Extensors?

**DOI:** 10.3390/jfmk9020073

**Published:** 2024-04-12

**Authors:** Eleftherios Kellis, Athanasios Konstantopoulos, Georgios Salonikios, Athanasios Ellinoudis

**Affiliations:** Laboratory of Neuromechanics, Department of Physical Education and Sport Sciences at Serres, Aristotle University of Thessaloniki, TEFAA Serres, Agios Ioannis, 62100 Serres, Greeceellinoud@phed-sr.auth.gr (A.E.)

**Keywords:** isokinetics, muscle length, pelvis, anterior, posterior, hamstrings, strength imbalance

## Abstract

The purpose of this study was to examine the effect of pelvic tilt angle on maximum hip and knee muscles’ strength and antagonist/agonist strength ratios. Twenty-one young males and females performed maximum isokinetic concentric knee extension–flexion and hip extension–flexion efforts at 60°·s^−1^, 120°·s^−1^, and 180°·s^−1^ from three positions: anterior, neutral, and posterior pelvic tilt. Peak torques and knee flexor-to-extensor and hip flexor-to-extensor torque ratios were analyzed. An analysis of variance showed that peak hip extensor torque was significantly greater in the anterior pelvic tilt condition compared to either neutral or posterior pelvic tilt angles (*p* > 0.05). No effects of changing pelvic tilt angle on hip flexor, knee flexor, or knee extension values were found (*p* > 0.05). The hip flexor-to-extensor torque ratio decreased (*p* < 0.05) in the anterior pelvic tilt position relative to the other positions, while no difference in the knee flexor-to-extensor ratio between pelvic positions was observed (*p* > 0.05). This study shows that an increased anterior pelvic tilt affects the maximum isokinetic strength of the hip extensors, supporting previous suggestions regarding the link between pelvic position and hip and knee muscle function. Isokinetic testing from an anterior pelvic tilt position may alter the evaluation of hip flexion/extension strength.

## 1. Introduction

The hamstrings are bi-articular muscles that act as hip extensors and hip flexors. The hamstrings play an important role in various athletic movements as they control the movement of the hip and knee, e.g., when sprinting or kicking [1]. Hamstring muscle function has been associated with an altered anterior cruciate ligament loading [2], while injuries to the muscles themselves are very frequent in athletes, especially soccer players and sprinters [3]. For these reasons, the evaluation of isokinetic knee flexor and hip extensor maximum strength for the conditioning, prevention, and rehabilitation of various knee injuries has been extensively studied [4,5,6,7].

The isokinetic strength of the hip and knee muscles varies with body position due to changes in the length, moment-arm, and activation of the involved musculature. Although changes in hip and knee angle predominately influence isokinetic hamstring strength [1], there is evidence that there is an association between pelvic position and hamstring function [8,9,10]. First, from an anatomical point of view, the long head of the biceps femoris is connected to the sacrotuberous ligament, whereas all hamstrings attach to the ischial tuberosity [8]. A systematic review of the evidence showed that increased hamstring tension limits pelvic mobility and lumbar motion [9]. For this reason, strategies to reduce hamstring stiffness have been proposed that are beneficial for increasing pelvic mobility and may perhaps assist in reducing lower back pain [11]. Research examining the mechanisms of hamstring injuries has also found that there is an association between hamstring injury and an elevated anterior pelvic tilt [12,13,14]. This finding was attributed to the association between pelvic mobility and hamstring length during sprinting, which is the most frequent mechanism of injury [12,13,14]. Research has also reported that restricting pelvic motion decreases the activation of the hip extensor muscles and increases isokinetic trunk strength [15]. The effect of pelvic position on hamstring function has only been examined by Nakamura et al. [16], who found that at the same hip joint angle, the passive tension applied to the hamstring muscles is greater in the anterior pelvic tilt than in neutral and posterior tilt positions. Moreover, in the same study, the passive knee flexion torque increased significantly with an anterior tilt of the pelvis. To the best of our knowledge, although pelvic position may influence the length and mechanical properties of the hamstrings, the influence of pelvic tilt on the ability of this muscle group to generate force remains unclear.

Isokinetic testing often involves the evaluation of strength balances between the knee flexors and extensors as well as the hip flexors and extensors [5]. It is known that changes in the hip and knee flexion angle influences the strength values of both antagonistic muscle groups [17]. So, if pelvic tilt influences the maximum force generation capacity of one or both antagonistic muscle groups, then one may expect that this would have an effect on the antagonist-to-agonist strength ratios as well.

From an anatomical standpoint and based on the above literature findings, it seems logical to expect pelvic tilt to affect the length and force generation capacity of the involved musculature. No research, however, has previously examined the effect of pelvic tilt on knee flexor and hip extensor muscle strength. Such information can assist in better explaining the role of pelvic tilt for maximum strength capacity as well as to improve isokinetic testing protocols by measuring the pelvis position during the test. The purpose of this study was to examine the effect of pelvic tilt angle on maximum strength and antagonist/agonist strength ratios during isokinetic tests of the extensors and flexors of the hip and the knee. It was hypothesized that pelvic tilt would affect the hip extensor and flexor strength. Moreover, it was hypothesized that pelvic tilt would influence the knee flexor strength, but not knee extensor strength. Further, it was expected that the pelvic tilt would have a significant impact on the knee flexor-to-extensor (KF/KE) and hip flexor- to-extensor (HF/HE) strength ratio.

## 2. Materials and Methods

### 2.1. Design

The participants attended three sessions on separate days. In session 1, the participants were familiarized with isokinetic testing, while hip extension/flexion and knee flexion/extension were assessed in sessions 2 and 3, respectively. In each session, the maximum isokinetic torque was measured at three different pelvic tilt angles.

### 2.2. Participants

Twenty-one healthy participants, of which thirteen were males (age 21.1 ± 1.1 years; height, 1.80 ± 0.07 m; body mass, 76.7 ± 5.2 kg) and eight were females (age 21.2 ± 0.6 years; height, 1.69 ± 0.06 m; body mass, 66.2 ± 9.4 kg), volunteered to take part in this investigation. To be included in the study, the athletes had experienced no musculoskeletal injury for the past year prior to the measurement session. Each participant completed a pre-exercise health questionnaire and signed a written informed consent document. All participants were amateur athletes engaging in sports (soccer, basketball, track-and-field, handball, tennis, and volleyball) training 4 times a week. This study was approved by the university’s institutional ethics committee (ERC03-2024). All strength tests were performed on a Humac Norm isokinetic dynamometer (Humac Norm, Cybex CSMI, Stoughton, MA, USA).

### 2.3. Procedures

**Figure 1:**
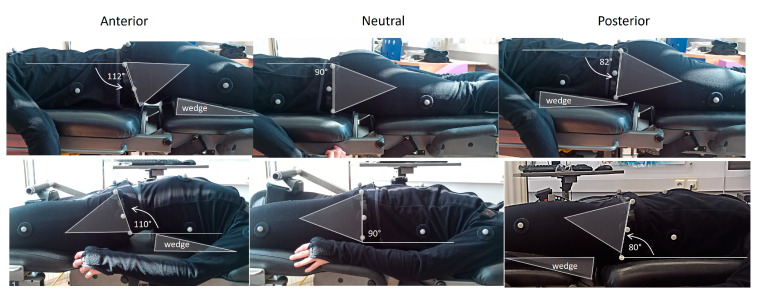
Illustration of the three pelvic tilt positions from the prone position, which was used for knee strength testing, and from the supine position which was used for hip strength testing. Markers were placed at the anterior and posterior superior iliac spines and the angle between the lines connecting these two markers relative to the horizontal was defined as the pelvic tilt angle. To better illustrate the change in pelvis position in each image, a triangular shape was superimposed so that one side coincided with the line connecting the anterior and posterior superior iliac spines. Similarly, to show the position of the body in each testing condition, a triangle shape was superimposed on the wedge, which was inserted underneath the thigh or the trunk area to increase the pelvic tilt angle during isokinetic testing.

The isokinetic testing of the right leg hip and knee muscles was performed from three different pelvic tilt conditions: neutral, 8–12° anterior pelvic tilt and 8–12° posterior pelvic tilt (Figure 1). Neutral was considered the position of the pelvis when the participant was in the supine (for hip muscle strength tests) and prone position (for knee muscle strength testing). In the neutral position, the pelvis is tilted about 3–4° anteriorly and posteriorly for the supine and prone positions, respectively. To achieve anterior and posterior pelvic tilt, a customized adjustable pad was used. To standardize the pelvic position, reflection markers were placed on the anterior iliac and posterior iliac spines and secondary markers along the thigh and the trunk. Then, digital photographs with a camera (ELP, Elp-usbg1200p01-MFV, Shenzhen Ailipu Technology Co., Ltd., Shenzhen, China, sampling rate 90 Hz, global shutter) placed perpendicularly to the body were obtained while the participant performed a submaximal contraction. The pelvic tilt position was measured as the angle between the lines connecting the anterior superior iliac spine and the posterior superior iliac spine and the horizontal.

Hip extension/flexion measurements of the right leg were obtained from the supine position (Figure 1). The dynamometer pad was placed at the lower end of the tested thigh about 5 cm from the lateral femoral condyle with the dynamometer axis aligned with the greater trochanter. Straps were used to stabilize the pelvis and the trunk while the participants were instructed to cross their arms over their chest during testing. Following the manufacturer’s guidance, the weight of each participants’ limb was measured at 60° hip flexion angle and it was used to correct the recorded torque for the effects of gravity. The range of motion was from 0° (neutral hip angle) to 90° of hip flexion. Before the isokinetic testing, a standard warm-up consisting of 15 min of cycling and 10 min of hamstring and quadricep static stretching exercises was performed. After taking the testing position, the protocol started with 1 set of 5 submaximal hip extension and flexion efforts at 120°·s^−1^ in each pelvic tilt condition. The main testing protocol included the performance of 5 concentric hip extension and flexion efforts with the knee flexed 90°, at angular velocities of 60°·s^−1^, 120°·s^−1^, and 180°·s^−1^. The subjects were instructed to exert maximal effort through the whole range of motion during the test. A time interval of 5 min was provided between different pelvic tilt positions and a 1 min interval was used between different sets. The sequence of tests was randomized across the types of angular velocities and pelvic tilt angles.

The assessment of knee flexors and extensor strength was performed from the prone position (Figure 1) using the same protocol as that of the hip extensors and flexors. The knee range of motion was set from 0° (full extension) to 90° of knee flexion. Prior to the test, the limb was weighed at 30° using the static gravitational correction procedure recommended by the manufacturer.

### 2.4. Data Analysis

Of the five repetitions, the repetition with the gravity-corrected maximum joint torque was analyzed. The range of motion was restricted from 10° to 80° to avoid inertial effects. The peak torque was determined from the raw torque and angular position data and used for further analysis. The KF/KE and HF/HE ratios were also calculated in each pelvic test position.

Thirteen participants were retested one week after the testing sessions to determine the reliability of the measurements.

### 2.5. Statistical Analyses

Statistical analysis was performed using the Statistical Package for the Social Sciences (v 29.0. IBM Corp., Armonk, NY, USA). Normal distribution was checked using Shapiro–Wilk tests. The test–retest reliability (ICC_2,1_) was examined by calculating the ICC and the 95% confidence interval (CI: 95%) [18].

Four-way analysis of variance (ANOVA) designs were used to examine the differences in either hip or knee maximum torque between different pelvic tilt angles (anterior, neutral, posterior), angular velocities (60, 120, 180°·s^−1^), muscle groups (extensor, flexor), and sex (males, females). Differences in HF/HE and KF/KE ratios between pelvic tilt angles at three angular velocities (30, 240°·s^−1^) were examined using a three-way ANOVA. Effect sizes were also calculated using the partial eta squared (η^2^) values [19]. Significant F values were followed by Tukey’s post hoc tests to determine the significance between group means. The level of significance was set at *p* < 0.05.

## 3. Results

The examination of the ANOVA findings showed that there were non-significant interaction effects between sex and pelvic tilt angle on all variables. Hence, the data were pooled across the sexes.

### 3.1. Reliability Measurements

The ICC results for the reliability of the hip strength and HF/HE ratios are presented in Table 1. The calculated ICC ranged from 0.65 to 0.95, indicating high reliability [18]. The mean (±standard deviation) group values and the results from the ANOVA comparisons, which were used to determine the ICC, are presented in Supplementary Table S1.

The ICC results for the reliability of the strength and HF/HE ratios are presented in Table 2. The calculated ICC ranged from 0.69 to 0.98, indicating high reliability [18]. The mean (±standard deviation) group values and the results from the ANOVA comparisons, which were used to determine the ICC, are presented in Supplementary Table S2.

### 3.2. Maximum Torque Values

The mean hip extension and flexion torque values are presented in Table 3. The ANOVA indicated a non-significant interaction effect of pelvic tilt, angular velocity, and muscle group (*p* > 0.05). There was, however, a statistically significant pelvic tilt by muscle group effect (F_2,40_ = 8.72, *p* = 0.0001, η^2^ = 0.324). Post hoc Tukey tests indicated that the hip extension torque (averaged across all angular velocities) with the anterior pelvic tilt angle was significantly greater than the torque at the other two angles (*p* < 0.05). In contrast, no pelvic tilt angle effect on hip flexion torque was found (*p* > 0.05). Finally, there were significant main effects for muscle group (F_1,19_ = 55.96, *p* = 0.0001, η^2^ = 0.774), as hip extensor torque was greater than hip flexion torque (*p* < 0.05), and for angular velocity (F_2,38_ = 175.01, *p* = 0.0001, η^2^ = 0.902), as torque decreased from 60°·s^−1^ to 180°·s^−1^ (*p* < 0.05).

The mean knee extension and flexion torque values are presented in Table 4. The ANOVA indicated a non-significant interaction effect of pelvic tilt, angular velocity, and muscle group (*p* > 0.05). The main effect for pelvic tilt was also non-significant (*p* > 0.05). There were significant main effects for muscle group (F_1,19_ = 99.17, *p* = 0.001, η^2^ = 0.839), as knee extension torque was greater than knee flexion torque (*p* < 0.05) and for angular velocity (F_2,38_ = 39.80, *p* = 0.001, η^2^ = 0.677), as torque decreased from 60°·s^−1^ to 180°·s^−1^ (*p* < 0.05).

### 3.3. HF/HE and KF/KE Torque Ratios

**Figure 2:**
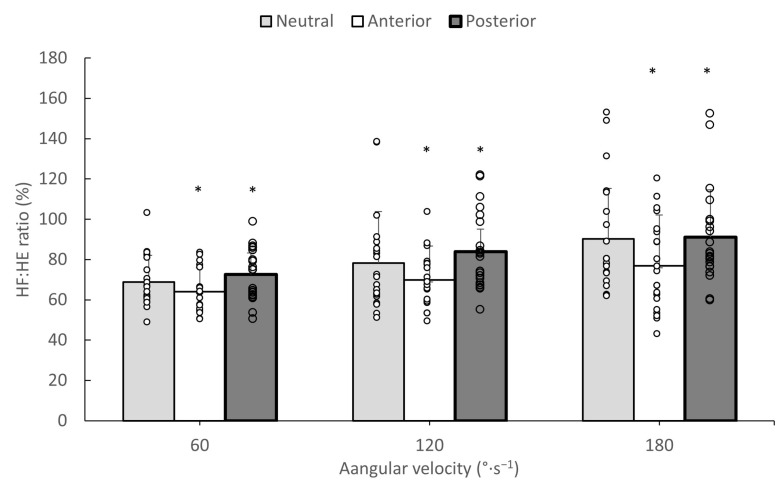
Mean hip flexor-to-extensor (HF/HE) torque ratios during isokinetic tests from three angular velocities in neutral, anterior, and posterior pelvic tilt angles. White dots indicate individual values and error bars indicate standard deviation (* significantly different compared to the other pelvic tilt angles at *p* < 0.05).

**Figure 3:**
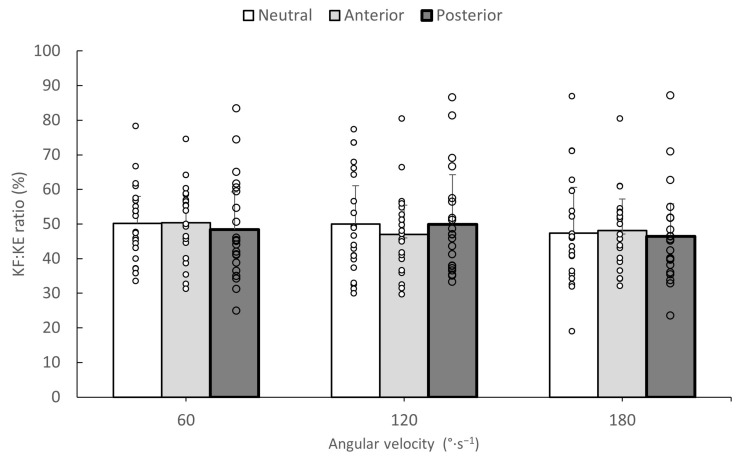
Mean knee flexor-to-extensor (KF/KE) torque ratios during isokinetic tests from three angular velocities in neutral, anterior, and posterior pelvic tilt angles. White dots indicate individual values and error bars indicate standard deviation.

The results for the estimated HF/HE and KF/KE ratios are presented in Figure 2 and Figure 3, respectively. The ANOVA indicated a non-significant pelvic tilt by angular velocity effect on HF/HE ratio (F_4,80_ = 0.72, *p* = 0.05, η^2^ = 0.035). Main effects were found for pelvic tilt (F_2,40_ = 9.35, *p* = 0.0001, η^2^ = 0.318) and angular velocity (F_2,40_ = 24.84, *p* = 0.0001, η^2^ = 0.552). Post hoc Tukey tests indicated that the HF/HE ratio was significantly lower in the anterior tilt position compared to the ratios of the other two pelvic tilt angles (*p* < 0.05). In addition, the HF/HE ratio in the posterior pelvic tilt angle was greater than the same ratio at the neutral pelvis position. The HF/HE ratio differed between angular velocities in the following order: 60°·s^−1^ < 120°·s^−1^ < 180°·s^−1^. (*p* < 0.05). For the KF/KE ratio, no interaction effects or main effects for pelvic tilt angle or angular velocity were found (*p* > 0.05).

## 4. Discussion

The main findings of this study were that anterior pelvic tilt caused an increase in hip extensor torque, but there were no effects on hip flexion, knee extension, or flexion torques. The HF/HE ratio was lower when the test was performed with anterior pelvic tilt than the other two angles, while posterior pelvic tilt led to a greater HF/HE ratio compared to neutral position tests. No difference in the KF/KE ratio between pelvic angles was found. To the best of our knowledge, the influence of pelvic tilt angle on maximum isokinetic torque has not been previously investigated.

The elevated hip extension torque when isokinetic testing is performed with an anterior pelvic tilt angle partly confirms the hypothesis of the study (Table 1). The differences in registered isokinetic torque between different initial pelvic tilt positions reflect changes in muscle function that are caused by changes in hip and pelvic tilt angles. During the isokinetic testing movement, the hip was first flexed from a neutral (starting) position to 90° (for hip flexion) and then the direction of the movement was reversed (for hip extension). It has been shown that when the hip extends, the pelvis tilts anteriorly [20]. This means that during a hip flexion of 90°, the pelvis is tilted posteriorly (a reduced lumbar lordosis) and when returning to the neutral position, the pelvis is tilted anteriorly. There are suggestions that when the pelvis tilts anteriorly, there are changes in the length and the moment-arms of the hip extensors, such as the hamstrings, the gluteus maximus, and the posterior head of the adductor magnus [21,22]. This means that the operative length of the involved musculature differs between the three pelvic tilt conditions. Hamstring length can increase during the forward rotation of the pelvis for two reasons: first, due to the increased tension of sacrotuberous ligament, which has a direct interface with the proximal tendon of the long head of biceps femoris [8] and, second, due to the simultaneous rotation of the proximal origin of the hamstrings (ischial tuberosity), which effectively increases the length of all hamstring components [23]. Based on anatomical descriptions of the line of the path of the hamstrings [21,23], it can be hypothesized that when the pelvis rotates forward relative to the femur, the hamstring line of force shifts away from the center of the hip rotation axis, thus increasing its moment-arm [22]. A greater anterior pelvic tilt may also influence the torque exerted by the gluteus maximus and the posterior head of the adductor magnus. To the best of our knowledge, there are no studies that have evaluated the mechanical properties of either of these muscles with changes in pelvic tilt angle. Research has shown that during submaximal prone hip extensions with the knee flexed, the pelvis is anteriorly tilted [24] and it is accompanied by a greater EMG activation of the gluteus maximus (~25% MVC) relative to the hamstrings (~15% MVC) [25]. Hence, an increased contribution of gluteus maximus to hip extensor force when the pelvis is tilted anteriorly is also possible. Finally, another possibility is that an anterior pelvic tilt increases the length and the moment-arm of the posterior head of the adductor magnus, which, like the hamstrings, attaches proximally to the ischial tuberosity and, distally, at several points along the linea aspera [21]. However, this is only an assumption, which, of course, needs to be verified by further research.

It has been suggested that shortened hip flexor muscles, such as iliopsoas or rectus femoris, may pull the pelvis anteriorly [26]. Hence, one would expect that a greater posterior pelvic tilt would increase hip flexion force due to an increase in its length. However, the present results showed no influence of pelvic tilt angle on maximum isokinetic hip flexion torque (Table 3). A previous study has reported no change in bi-articular rectus femoris muscle activation in various pelvic tilt positions, which may partly explain the present findings [27]. Clearly, more research is necessary to investigate the influence of pelvic tilt on the maximum strength capacity of the hip flexor muscles.

Changing the pelvic tilt angle did not have any influence either on knee flexor or knee extensor isokinetic strength (Table 4), which does not confirm the second hypothesis of the study. As already mentioned, based on previous suggestions [8,22], an increase in the anterior pelvic tilt shifts the working range of the hamstrings to longer muscle lengths. The present results, however, indicate that such an increase in length may not be enough to lead to a greater isokinetic knee flexion torque, at least when the test is performed from the prone position. In addition, it is unclear whether the changes in the line of force of the hamstrings when the pelvis tilts anteriorly or posteriorly can influence the moment-arm of these muscles about the knee joint axis. The effect of pelvic position on hamstring function was only examined by Nakamura et al. [16], who found that the passive knee flexion torque increased significantly with an anterior tilt of the pelvis. Given the absence of previous evidence, the present findings indicate that when active isokinetic strength is exerted, any changes in passive force development due to an altered pelvic position do not influence the overall isokinetic recorded knee flexion torque. Similarly, since the vastii muscles only act as knee extensors, a change in pelvic tilt can have minimal influence on the quadricep’s force/torque generation capacity as well.

The third hypothesis of the study was partly confirmed, as changes in pelvic tilt influenced the HF/HE torque ratio (Figure 3), but had no effect on KF/KE torque ratio (Figure 3), which makes sense, as a greater anterior pelvic tilt only increased the hip extension maximum torque (Table 3) and had no influence on other hip flexion or knee extensor or flexor torque values. Antagonist-to-agonist muscle strength ratios are often used for the evaluation of strength imbalances around a joint [5]. The present results imply that taking an anterior pelvic tilt position during isokinetic hip testing may shift the HF/HE ratios to lower values, while the opposite was observed when tilting the pelvis posteriorly. This may create additional inter-individual variability in the reported isokinetic ratios [5], which must be taken into consideration when designing isokinetic testing protocols.

The results of this study showed that the evaluation of isokinetic hip (Table 1) and knee (Table 2) torque from an anterior or posterior pelvic tilt position shows high test–retest reliability. To best of our knowledge, the reliability of isokinetic strength measurements using different pelvic tilt positions has not been previously examined. Placing the wedge underneath the thigh or the lumbar/abdominal area is critical to achieve a similar pelvic tilt position for all participants during the isokinetic test. This ensures a similar hip range of motion during the test, which ultimately determines the shape of the isokinetic torque–joint angular position curve.

The present results may help to clarify the influence of pelvic tilt on the function of the hip and knee flexors/extensors. In particular, the results partially confirm earlier studies in which a correlation between pelvic tilt and the function of the hamstrings was established [8,9,10]. Within the limitations of the present study, it appears that individuals who exert maximal isokinetic torque with increased anterior pelvic tilt are able to exert greater hip extension torque. One may speculate that this could be a strategy when athletes need to exert high hip extension torque when they perform movements such as sprinting or changing direction. This could help to explain why soccer players show a greater anterior pelvic tilt when they are fatigued [28]. Simultaneously, the sudden lengthening of the muscle–tendon unit due to excessive anterior pelvic tilt can increase the risk of hamstring injury [12,13,14] during sprinting and can also contribute to lower back pain [9].

There are limitations in this study. The results are applicable to healthy young males and females. Different results may be obtained in professional athletes or untrained individuals. Further, a standardized wedge was used to induce an anterior or posterior tilt in all individuals. Hence, the maximum range of motion of the participants in the anterior or posterior pelvic tilt was not measured and, therefore, the influence of flexibility on strength values was not examined. In addition, while every measure was taken to maintain a consistent and stable body position during isokinetic testing, movements of the thigh, pelvis, and trunk might have occurred, which might have had an impact on the results. Finally, the influence of trunk muscles, such as the lumbar muscle and the abdominals, on the recorded torque values was not examined.

## 5. Conclusions

Isokinetic testing from a greater anterior pelvic tilt only increased the torque of the hip extension and not the hip flexion, knee extension, or knee flexion torques. No changes in any of the isokinetic variables when the pelvis was tilted posteriorly were noted. The HF/HE ratio decreased, and the KF/KE ratio was unaltered in the anterior pelvic tilt position relative to the neutral or posterior tilt position. Future research could examine whether the elevated hip extension torque when the pelvic tilts anteriorly represents a strategy used by individuals to control hip motion during sport movements, such as sprinting. Further, isokinetic testing protocols should take into consideration the potential influence of an altered pelvic tilt position on hip strength values and antagonist/agonist muscle strength ratios.

## Figures and Tables

**Table 1:** Intraclass correlation coefficient (95% confidence intervals) resulting from comparing the test and retest hip strength values and the hip flexor-to-extensor (HF/HE) strength ratios (N = 13).

		Pelvic Tilt	
	Neutral	Anterior	Posterior
Hip extension			
60°·s^−1^	0.87 (0.63–0.96)	0.88 (0.67–0.96)	0.91 (0.75–0.97)
120°·s^−1^	0.93 (0.78–0.98)	0.88 (0.65–0.96)	0.94 (0.84–0.98)
180°·s^−1^	0.88 (0.66–0.96)	0.77 (0.13–0.71)	0.95 (0.86–0.98)
Hip flexion			
60°·s^−1^	0.89 (0.68–0.97)	0.88 (0.66–0.96)	0.87 (0.63–0.95)
120°·s^−1^	0.88 (0.66–0.96)	0.94 (0.81–0.97)	0.95 (0.85–0.98)
180°·s^−1^	0.93 (0.79–0.97)	0.94 (0.83–0.98)	0.89 (0.70–0.97)
HF/HE ratio			
60°·s^−1^	0.84 (0.54–0.95)	0.87 (0.64–0.96)	0.65 (0.19–0.87)
120°·s^−1^	0.91 (0.75–0.97)	0.93 (0.80–0.98)	0.94 (0.82–0.98)
180°·s^−1^	0.92 (0.77–0.97)	0.85 (0.59–0.95)	0.92 (0.77–0.97)

**Table 2:** Intraclass correlation coefficient (95% confidence intervals) resulting from comparing the test and retest knee strength values and the knee flexor-to-extensor (KF/KE) strength ratios (N = 13).

		Pelvic Tilt	
	Neutral	Anterior	Posterior
Knee extension			
60°·s^−1^	0.92 (0.76–0.97)	0.96 (0.89–0.99)	0.95 (0.86–0.98)
120°·s^−1^	0.95 (0.84–0.98)	0.98 (0.96–0.99)	0.94 (0.83–0.98)
180°·s^−1^	0.96 (0.88–0.98)	0.95 (0.85–0.98)	0.69 (0.24–0.89)
Knee flexion			
60°·s^−1^	0.91 (0.61–0.97)	0.94 (0.81–0.98)	0.82 (0.52–0.94)
120°·s^−1^	0.86 (0.40–0.96)	0.94 (0.71–0.98)	0.84 (0.57–0.94)
180°·s^−1^	0.88 (0.65–0.96)	0.87 (0.55–0.96)	0.69 (0.24–0.89)
KF/KE ratio			
60°·s^−1^	0.73 (0.35–0.91)	0.95 (0.83–0.98)	0.81 (0.51–0.94)
120°·s^−1^	0.75 (0.29–0.92)	0.91 (0.28–0.98)	0.92 (0.76–0.97)
180°·s^−1^	0.84 (0.47–0.95)	0.87 (0.48–0.97)	0.69 (0.25–0.99)

**Table 3:** Mean (±SD) group hip extension and flexion torque (Nm) values at three angular velocities and three pelvic tilt angles (* significantly different compared with neutral pelvic tilt values; ^ significantly different compared with other velocities; # significantly greater than hip flexion values, *p* < 0.05).

		Pelvic Tilt	
	Anterior	Neutral	Posterior
Hip extension			
60°·s^−1^	249.00 ± 72.07 *^#	231.90 ± 68.24 ^#	226.44 ± 78.24 *^#
120°·s^−1^	191.85 ± 59.35 *^#	178.66 ± 65.89 ^#	160.41 ± 64.44 *^#
180°·s^−1^	144.66 ± 61.38 *^#	131.09 ± 40.28 ^#	130.93 ± 56.96 *^#
Hip flexion			
60°·s^−1^	159.14 ± 53.01 ^	157.95 ± 50.52 ^	160.33 ± 54.44 ^
120°·s^−1^	131.42 ± 41.82 ^	131.04 ± 40.05 ^	133.12 ± 48.11 ^
180°·s^−1^	105.66 ± 40.77 ^	113.00 ± 39.28 ^	111.69 ± 46.72 ^

**Table 4:** Mean (±SD) group knee extension and flexion torque (Nm) values at three angular velocities and three pelvic tilt angles (^ significantly different compared with other velocities; # significantly greater than knee flexion values, *p* < 0.05).

		Pelvic Tilt	
	Anterior	Neutral	Posterior
Knee extension			
60°·s^−1^	156.85 ± 35.77 ^#	151.57 ± 48.92 ^#	159.53 ± 38.25 ^#
120°·s^−1^	130.95 ± 50.24 ^#	129.85 ± 51.43 ^#	125.00 ± 42.15 ^#
180°·s^−1^	109.49 ± 45.83 ^#	105.61 ± 44.84 ^#	110.19 ± 52.35 ^#
Knee flexion			
60°·s^−1^	78.44 ± 26.56 ^	74.61 ± 26.37 ^	71.38 ± 25.82 ^
120°·s^−1^	60.19 ± 22.48 ^	57.90 ± 20.13 ^	59.00 ± 18.76 ^
180°·s^−1^	50.00 ± 19.87 ^	47.38 ± 20.53 ^	47.00 ± 19.85 ^

## Data Availability

The data are available at Mendeley Data, V1, https://doi.org/10.17632/yvn5bmh4r3.1.

## Supplementary Materials

The following supporting information can be downloaded at: https://www.mdpi.com/article/10.3390/jfmk9020073/s1.
