# Supplementary material for: Does Pelvic Tilt Angle Influence the Isokinetic Strength of the Hip and Knee Flexors and Extensors?
Source: J Funct Morphol Kinesiol. 2024 Apr 12;9(2):73. doi: 10.3390/jfmk9020073 (PMC11036241; doi:10.3390/jfmk9020073)
Supplement: Supplementary file 1 [file jfmk-09-00073-s001.zip › TABLE SUPPLEMENTARY 1.pdf]

Table S1: Mean (± standard deviation) maximum hip extension and flexion torques and hip flexion-to-extension (HF/HE) torque ratios at three angular velocities during day 1 (test) and day 2 (retest) measurements which were used to estimate the intraclass correlation coefficients (ICC) for each variable. F-ratios (df = degrees of freedom) and the level of significance (p) indicate whether the estimated ICCs are statistically significant.

	Anterior Pelvic Tilt			Neutral			Posterior Pelvic Tilt		
	Day 1	Day 2	F-ratio, df = 12	Day 1	Day 2	F-ratio, df = 12	Day 1	Day 2	F-ratio, df = 12
Hip extension									
$60°\cdot s^{-1}$	247.07 ± 75.03	252.45 ± 99.02	15.84, p< 0.001	228.00 ± 77.32	232.36 ± 86.84	13.73, p< 0.001	217.72 ± 82.03	222.04 ± 86.06	21.58, p< 0.001
$120°\cdot s^{-1}$	196.30 ± 64.84	197.05 ± 69.33	14.78, p< 0.001	181.48 ± 64.94	185.67 ± 80.95	25.62, p< 0.001	162.96 ± 88.81	164.33 ± 92.44	35.21, p< 0.001
$180°\cdot s^{-1}$	148.53 ± 74.11	155.19 ± 79.10	46.71, p< 0.001	141.07 ± 54.11	140.89 ± 59.36	14.83, p< 0.001	135.10 ± 79.12	134.77 ± 73.83	40.31, p< 0.001
Hip flexion									
$60°\cdot s^{-1}$	160.38 ± 61.91	158.28 ± 58.38	15.05, p< 0.001	158.68 ± 57.43	158.86 ± 59.97	16.39, p< 0.001	160.92 ± 62.82	156.17 ± 64.45	13.21, p< 0.001
$120°\cdot s^{-1}$	131.38 ± 50.35	131.59 ± 48.80	29.61, p< 0.001	130.53 ± 47.36	131.92 ± 56.53	15.21, p< 0.001	132.43 ± 59.32	130.40 ± 55.54	38.83, p< 0.001
$180°\cdot s^{-1}$	108.76 ± 48.97	104.31 ± 49.17	32.63, p< 0.001	114.38 ± 48.42	110.66 ± 47.83	27.53, p< 0.001	113.20 ± 55.50	112.67 ± 54.48	17.45, p< 0.001
HF/HE ratio									
$60°\cdot s^{-1}$	64.32 ± 11.92	63.93 ± 12.04	13.93, p< 0.001	66.97 ± 8.94	68.68 ± 8.32	10.43, p< 0.01	74.16 ± 12.48	71.15 ± 13.60	4.63, p< 0.03
$120°\cdot s^{-1}$	67.21 ± 11.96	67.94 ± 14.38	28.27, p< 0.001	72.45 ± 16.36	74.72 ± 22.76	21.22, p< 0.001	85.92 ± 17.53	86.98 ± 20.68	32.28, p< 0.001
$180°\cdot s^{-1}$	76.63 ± 23.22	70.54 ± 22.34	14.07, p< 0.001	82.92 ± 21.43	80.39 ± 18.85	24.42, p< 0.001	87.79 ± 13.48	86.94 ± 14.90	23.55, p< 0.001
