# Supplementary material for: Does Pelvic Tilt Angle Influence the Isokinetic Strength of the Hip and Knee Flexors and Extensors?
Source: J Funct Morphol Kinesiol. 2024 Apr 12;9(2):73. doi: 10.3390/jfmk9020073 (PMC11036241; doi:10.3390/jfmk9020073)
Supplement: Supplementary file 1 [file jfmk-09-00073-s001.zip › TABLE SUPPLEMENTARY 2.pdf]

Table S2: Mean (± standard deviation, N = 13) maximum knee extension and flexion torques and knee flexion-to-extension (KF/KE) torque ratios at three angular velocities during day 1 (test) and day 2 (retest) measurements which were used to estimate the intraclass correlation coefficients (ICC) for each variable. F-ratios (df = degrees of freedom) and the level of significance (p) indicate whether the estimated ICCs are statistically significant.

	Anterior Pelvic Tilt			Neutral			Posterior Pelvic Tilt		
	Day 1	Day 2	F-ratio, df = 12	Day 1	Day 2	F-ratio, df = 12	Day 1	Day 2	F-ratio, df = 12
Knee extension									
$60°\cdot s^{-1}$	159.61 ± 44.44	162.32 ± 43.61	57.41, p< 0.001	161.89 ± 56.72	165.23 ± 47.56	22.93, p< 0.001	156.92 ± 45.13	154.84 ± 36.25	39.84, p< 0.001
$120°\cdot s^{-1}$	134.23 ± 57.84	133.23 ± 53.75	135.97, p< 0.001	133.07 ± 56.36	134.76 ± 47.01	34.13, p< 0.001	130.84 ± 46.24	127.76 ± 37.35	34.15, p< 0.001
$180°\cdot s^{-1}$	110.53 ± 50.01	110.41 ± 40.45	37.94, p< 0.001	111.38 ± 40.55	112.39 ± 47.02	50.96, p< 0.001	111.53 ± 56.13	100.31 ± 41.29	6.75, p< 0.02
Knee flexion									
$60°\cdot s^{-1}$	77.84 ± 29.77	82.07 ± 28.86	35.31, p< 0.001	77.23 ± 31.27	85.15 ± 28.47	30.22, p< 0.001	74.46 ± 29.30	80.23 ± 23.99	10.43, p< 0.001
$120°\cdot s^{-1}$	59.69 ± 25.25	65.07 ± 23.83	47.65, p< 0.001	57.39 ± 23.29	65.00 ± 18.97	22,67, p< 0.001	61.46 ± 18.91	63.69 ± 17.03	10.84, p< 0.001
$180°\cdot s^{-1}$	50.07 ± 20.68	56.15 ± 20.33	20.31, p< 0.001	48.78 ± 22.49	51.69 ± 21.41	18.30, p< 0.001	46.07 ± 21.63	45.38 ± 16.89	5.75, p< 0.001
KF/KE ratio									
$60°\cdot s^{-1}$	49.32 ± 12.95	51.21 ± 13.19	43.26, p< 0.001	48.67 ± 12.93	52.40 ± 13.73	6.91, p< 0.02	49.16 ± 17.38	53.61 ± 16.59	10.99, p< 0.001
$120°\cdot s^{-1}$	45.99 ± 15.04	53.22 ± 15.04	49.17, p< 0.001	44.68 ± 14.57	50.44 ± 12.32	9.65, p< 0.01	51.30 ± 18.72	52.88 ± 16.93	23.53, p< 0.001
$180°\cdot s^{-1}$	48.69 ± 13.14	53.02 ± 13.07	23.35, p< 0.001	43.43 ± 16.53	48.77 ± 15.52	16.15, p< 0.001	45.32 ± 16.36	48.53 ± 18.07	5.28, p< 0.03
